# The Development of an RFID Solution to Facilitate the Traceability of Patient and Pharmaceutical Data

**DOI:** 10.3390/s17102247

**Published:** 2017-09-29

**Authors:** María Martínez Pérez, Guillermo Vázquez González, Carlos Dafonte

**Affiliations:** 1 Department of Information and Communications Technologies, Faculty of Computer Science, Campus Elviña S/N, University of A Coruña, E-15071 A Coruña, Spain; dafonte@udc.es; 2 Complejo Hospitalario Universitario de A Coruña (CHUAC), 15006 A Coruña, Spain; guillermo.vazquez.gonzalez@sergas.es

**Keywords:** hospital, pharmacy, intravenous mixtures, radiofrequency identification

## Abstract

One of the principal objectives of hospitals is to increase the quality of care of the patient. This is even more of a priority in Day Hospitals where certain medication requires special attention, from its preparation in the Pharmacy service to its delivery to the patient in the Day Hospital. In the case of expensive medicines, nursing staff have to comply with very detailed instructions in their administration to the patient (name of medicine, route, dosage, schedule, previous medication, conditions of conservation, etc.). This work focuses on the development of a multi-faceted hub application to facilitate the traceability of mixed intravenous medication from the beginning to the end of the process of prescription–validation–dosing–preparation–administration (PVD-PA) and be available to all health professionals involved: doctors, pharmacists, and the nursing staff of the Hospital Pharmacy and Day Hospital.

## 1. Introduction

Nowadays, all of the activities involved in the care process of the patient are important. This translates into a chain of tasks in which each one is a vital link in the protection of the patient.

The smooth functioning of the care process is carried out to improve the overall safety of the patient, which is considered to be one of the most important objectives of the Spanish Health Service (SNS). Methods are required to avoid unnecessary harm to the patient. Aspects of this work which facilitate compliance to safety standards will be discussed subsequently in this article [1].

At present, intense research into patient safety is being carried out, the evidence of which is reflected in the range of scientific articles being published in health-related publications. Nevertheless, valid and precise evidence is required to evaluate the actions that are being carried out to increase patient safety in hospitals.

The law 29/2006 regulating the use and rationale of medication and health products obliges their traceability but does not define what this means. According to AECOC, traceability refers to those pre-established procedures that allow for the history, route, lot number and/or series no., and whereabouts of a product to be known at any given time, using specific tools [2]. The objective, more specifically, is to identify the entire route of a medicine from its reception and elaboration in a health service department to its eventual administration to the patient.

These concerns are even more important in the case of those medicines that are used to treat pathologies and involve a high level of toxicity where errors could result in the death of the patient. It is necessary to have a secure and automatic diagnosis and prescription to eradicate human error. This safety should be maintained by pharmacy personnel during the process of prescription–validation–dosing–preparation–administration (PVD-PA) and by the nursing staff of the Day Hospital in the final administration of the medicine.

It is important to integrate all the necessary information into the same software (SW). It is not only about having a centralized database (which avoids repetition and possible human error) but also about having available in the same application all those tasks to be carried out by the different professional profiles:

- The doctor prescribes the intravenous mixture for the patient.
- The pharmacist validates the prescribed medication for the patient (that the name, dosage, route, instructions, and other data are correct).
- The doctor prescribes the upcoming administration of the intravenous mixture (the date, e.g., 18 July 2017).
- The patient arrives at the hospital (presents himself/herself at the Day Hospital at the time programmed by the doctor). The subject is located in real time and accurately identified by the radio-frequency identification (RFID) tag [3,4,5,6].
- The doctor confirms that the state of health of the patient is adequate for the administration of the medication (e.g., that there is no fever, and that any required medication has been administered prior to the medication currently prescribed (the most common being paracetamol)).
- The doctor advises the Pharmacy Service that the patient has arrived and that the medication should be prepared. This must be carried out in real time to avoid a situation where the patient cannot receive the prepared medicine. Imagine the situation where the medicine is prepared and the patient cannot receive it or has already left, resulting in a very expensive loss. It is necessary to prepare the intravenous mixture in real time, since the stability of the medication is very short and has to be administered to the patient within a brief period of time.
- The doctor has to certify that the patient is not well enough or is not prepared to receive the medication in order to postpone the administration.
- The nursing personnel of the Pharmacy Service initiate the preparation of the medication in line with the instructions of the pharmacists (dilution, reconstitution of the vial, etc.).

In the described circuit, the only difference between the situation before and after the implementation of our work consists of the tools that were applied (changing from paper, etc., to the described innovative system).

The role of Information and Communication Technologies is particularly relevant given that the integration of technology such as RFID can contribute significantly to the development of these tasks. In comparison with other identification technologies, they improve safety and efficiency in their capacity to precisely and unambiguously identify patients and/or elaborated intravenous mixtures and their components (name, lot n°, expiry date). In the case of a medicine alert, the registration of this data allows for immediate knowledge (almost in real time) concerning the patient to whom a specific lot nº of medicine has been administered.

Other identification technologies such as QR or barcode do not comply with all the requirements of this project (although they are to be recommended for other scenarios). A primary reason for this is that they do not allow for the localization of patient or medication in real time and at distance, or of hundreds of tags simultaneously, without there being a direct line of vision between the tag and the reader.

This advantage of RFID is particularly useful in the event of consultation or of patients going astray because it eliminates the need for internal telephone calls and allows health personnel to attend to their highly specialized duties.

The inability to record tracking in real time does not allow for the subsequent analysis and exploitation of data to measure waiting and transport times, detect bottle necks, and thereby improve the efficiency of the service. The use of barcodes or QR would necessitate a study of origin and destination which would not be out of place in another service such as emergency services [7,8], but in this case, involving medication of high cost and risk, the suggested tracking is necessary and justified. This coincides with the expressed needs of healthcare staff at the University Hospital of A Coruña (CHUAC) and the recommendations of the Ministry of Health and Social Services [1].

It is indeed quite a challenge to develop an innovative project incorporating RFID that is implemented into a real-life environment and which, at the same time, complies with the necessary requisites to be used in daily clinical practice in a hospital complex [9,10].

The following sections describe the main objectives and benefits of this work, the methodology that was applied, the development, and finally, we discuss and detail the main conclusions that could be reached.

## 2. Objectives and Benefits

This work forms part of the PI10/02442 Research Project funded by the Carlos III Institute of Health, the aim of which is to improve the PVD-PA process of medication for patients in the Day Hospital and Pharmacy Service of CHUAC [11,12,13]. This means that it is attempting to obtain the traceability of medication developed by the Pharmacy Service that is considered to be expensive and of high risk in its administration to the patient. These characteristics justify undertaking methods to improve the control of intravenous mixtures from their origin in the Pharmacy Service, during their transport, until their subsequent administration to the patient in the Day Hospital.

Integrating RFID technology into this process and automating it is highly complex and innovative because, until now, this has only been done on paper. In addition, this work requires highly specialized knowledge which is dispersed in different medical units (Pharmacy, Rheumatology, Dermatology, Pediatric, Day Hospital, and Neurology services, etc.) and, at the same time, available in diverse information media (the corporate software of SERGAS, SILICON, IANUS, laboratory, medication technical indices, vademecum, etc.).

Prior to the development of this work, efforts were made to acquire software of similar characteristics that would adjust to the needs of health personnel and that would comply with the requisites previously specified, but none could be found in the market. Pharmaceutical Laboratories have also been consulted for the development or acquisition of similar software but, again, to no avail. This research work corresponds to latent needs in the daily practice of the hospital.

Even though projects with similar functionalities exist (regarding location and/or identification of sanitary elements), one of the main difficulties of the RFID technology is that it requires the tailored selection of the components (labels, readers, software) for the place of implementation. Indeed, a concrete detail such as the material of the walls may influence the correct functioning of the RFID system.

Existing projects that could be detected [14,15,16,17,18,19,20,21,22,23] are not focused on the same type of medication and/or traceability (registration of preparation date, exit from the Pharmacy Service, arrival at the Day Hospital, hour of delivery and administration, etc.), and as such do not provide the same traceability details.

For instance, the medication that was included in this work consists of liquids in plastic containers, which are typically enemies of RFID; what makes our project unique and highly innovative is that we are nevertheless able to make flawless readings thanks to the labels (which are adhered like little flags) and the hardware (HW) that was specifically developed for us by specialized companies.

Another distinguishing feature is the reading distance: the double frequency labels can be read at a short distance by the mobile phones of the nursing staff (while reading labels of other patients/drugs when these are nearby), and at the same time at a long distance by the RFID transporting cart, using the RFID label that records more traceability data. Patients are located through their active WIFI tag; the localization motor has a calculation error between 1 and 4 m for the position of the patient or cart, but is able to locate the patients or RFID cart even if these are located at a maximum distance of approximately 50 m from the access point.

The RFID technology referred to in this work is a key tool in obtaining the traceability of medication from its preparation, transport, and eventual administration to the patient in the Day Hospital (time and date of preparation; time and date of delivery; time and date of administration; lot nº and date of the components and prepared medication; location in real time; data relating to the patient, the pharmacist, or nursing personnel; medication conditions for stability, conservation, and administration and medication necessary to administer beforehand; route; dosage; guidelines for use, etc.).

To integrate this software into a health setting is a pioneering proposal and even more so when the setting is a Day Hospital; the design of all the hardware components for this project has been specific and tailor-made by a Spanish firm specializing in RFID components.

This project requires a hub for clinical information from the moment the doctor prescribes to the eventual administration of the medicine by the nurse to the patient (location, lot nº, date of expiry, transport conditions, conservation, administration, etc.). The principle benefits of this work will be to increase the quality of care received by the patient given that RFID clearly identifies the patient and prescribed medicine and assures that nursing personnel will administer the prescribed medicine in the best conditions. Other health users can, similarly and in real time, locate nurses and medication, detect bottlenecks, and contribute notably to the overall efficiency of the service.

The objective of this article is (1) to present a case study of a hospital that has developed an RFID system for the management of intravenous mixtures; and (2) to propose a design of a solution that allows for tracking and matching patient/intravenous mixtures that are administered in the Day Hospital in the University Hospital of A Coruña (CHUAC).

The work is not centred on any intravenous mixture but is focused on those considered to be expensive and of high risk in their administration to the patient. A dose can reach 3000 euros in price (the majority of patients come to the hospital on a monthly basis) and is prepared according to the pharmotherapeutic profile of the patient (other parameters include the weight and the evolution of the pathology of the patient). Administering this medicine to another patient could result in their death and so justifies the existence of an application that facilitates and integrates all the tasks related to the prepared mixture.

The precise identification of the prepared intravenous mixture is vital. To do this, an RFID tag is used which has been tailor-made for this project by a Spanish company specializing in RFID. This tag has two components: a near-field communication (NFC) chip (that reads the mobile of nursing personnel before the administration of medication to the patient) [12] and an ultra-high frequency (UHF) chip (which reads the RFID cart during transport of the medication from the Pharmacy Service to the Day Hospital) facilitating its location in real time.

In addition, this UHF chip can be read by the tray which is situated in the Day Hospital. The orderly, on arrival at the Day Hospital with the cart, deposits the intravenous mixtures in the RFID tray which also registers the delivery time.

The nursing staff of the Pharmacy Service match the unique identifier (UID) of the RFID tag with the patient´s information and the associated medication. To do this, they have to pass the RFID tag of the medication by the RFID readers which are connected to the computer using USB.

The last step requires the Day Hospital nursing personnel administering the medication to the patient which involves them reading the RFID tag of the medication and subsequently the patient´s tag to confirm that they are administering the correct medicine in the required administration conditions [12].

The aim is to implement an application that complies with the aforementioned tasks, that is capable of computerizing a highly complex and specialized process (until now, only described on paper), and that can integrate all the health personnel involved. The nursing staff of the Pharmacy Service, when they are preparing the intravenous mixture, can register, at the same time, the lot nº and the expiry date of the components as well as the details of the prepared medicines. This means, in the case of a medication alarm, that patients who have been treated with a specific lot nº batch, can be located almost in real time.

An additional advantage of the project is that all the information required by the doctor, pharmacist, or nursing staff of the Pharmacy Service (whether related to the patient, medication, origin or destination of the service, etc.) is integrated into the application.

This provides safety and strengthens the entire process. There is no possibility of health professionals handling external information (uncontrolled or out of place) given the extensive work carried out during the analysis phase when all the essential data was integrated into this software. This objective also reinforces the safety of the process (avoiding possible adverse events) and the sustainability of the Public Health System (by avoiding the possibility of administering unauthorized or out-of-date doses).

To integrate RFID technology into this highly specialized environment is indeed a challenge. A number of different functionalities were considered for research (to evaluate financial and technical viability) which were eventually resolved:

- The possibility of reading double frequency tags on liquid. The majority of intravenous mixtures are diluted in serum (this was resolved by attaching the tag in a flag position to the intravenous mixture) [12].
- 100% reading of the intravenous mixtures while being transported in the RFID cart.
- Location, in real time, of the RFID cart (using an active WIFI RFID tag) and the intravenous mixture content.
- The implementation of RFID Systems that have three different operating frequencies: passive tag UHF and NFC for medication, access points (fixed RFID WIFI readers), tray (fixed UHF reader), mobile (NFC mobile reader), patient’s active WIFI tag, NFC USB reader, and a UHF USB reader. The medication developed in the Pharmacy Service is labelled with an RFID tag (with both UHF and NFC frequencies), and nursing personnel relate the UID in the identification tag to the binominal patient–medication through USB readers connected to computers located in the medication area.
- Design of a protocol for working in a cabin to prepare this type of medication and compatible with the handling of RFID components.
- RFID printer to print RFID visual tags with the design that has been used until now.

These medications are expensive, high-risk treatments and are administered to patients in the Rheumatology, Dermatology, and Pediatric services; registration of their prescriptions using this tool has been in use since 2014 [12,13].

An additional objective of this project is to achieve the use of the application with minimal difficulty which means the training of health professionals and the development of the project in minimum time.

## 3. Methodology and Development

The present work should be considered as applied research that was carried out according to the Action Research methodology. The methodology that was applied follows the waterfall lifecycle model, with the analysis, design, implementation, and testing stages. Feedback is provided on previous stages. Feedback was required at the design stage when a doctor realized, after having seen the completed work, that a patient could simultaneously receive more than one active medicine, and therefore that the design carried out until that time was not compatible with the requisites.

To achieve the aforementioned objectives, the information relating to patient traceability and elaborated intravenous medication was integrated into an application [24] already in use in various CHUAC units.

The necessary stages for the development of a Protocol in the Day Hospital (PDH) are detailed below, and should respond to the needs of all users in line with the profile of specific health professionals (nursing personnel, doctors, and pharmacists) in the Pharmacy Service and Day Hospital.

### 3.1. Identify the Problem & Envision the Solution

The first stage in the development of the project involves the analysis. For this, it is necessary to revise the checklist of the application [24] in which the PDH is going to be integrated to check that it meets the prerequisites established in the project [12,13] by the health professionals.

A fundamental task involves reviewing the other implemented protocols to identify successful examples and determine how the problems might be resolved. While holding meetings with the hospital personnel, the documentation and legislation relating to adverse events at the centre should be reviewed in order to evaluate the most suitable techniques to reduce them [1].

The medications under consideration in this study are Abatacept, Tocilizumab, Remicade (Infliximab), and include biosimilars such as Inflectra (Infliximab) as they are the principal medicines involved in the research project to which this study belongs [12,13]. These medications are currently being prescribed by doctors using this PDH in the Dermatology, Rheumatology, and Digestive Health services. This PDH also allows for the subsequent exploitation of data to analyze the monthly and annual consumption of medication or the monthly/annual frequency of consumption by service. The PDH will minimize the possibility of human error and therefore improve the safety and efficiency of those services involved (accelerate the provision of services, and facilitate the analysis of defined indicators and the tracking of administered medication in the case of alert notifications).

### 3.2. Develop a Plan of Action

There are different parts to the development of the PDH:

- Record parameters (name, data type, modifiable or not, etc.)
- The main record
- Monitoring actions (grouped in tags)
- Rules (grouped in guides)
- Filters of data and daily clinical practice

Two types of rules exist:

- Obligatory: the value of the parameter is determined by the rule. Although the user may decide to change the value, it will return to the original one.
- Advisory: warning that the value is not recommended although another is permitted. The doctor, for example, can modify the previously recommended prescription.

Two different protocol designs were carried out, since doctors indicated that modifications were required in the original version.

The main difference between them is that prescription was included in the main registry of the patient. This means that medication was included in the main registry as soon as the patient registered in the Day Hospital.

The principal inconvenience with this is that if the patient changes medication (e.g., for reasons relating to the detection of another pathology, lack of effectiveness, or an adverse reaction to the treatment, etc.), this could give rise to problems in the registration or the subsequent use of data. It would then be necessary to include another main register for the patient to have other active biological treatment. Hence, in the first design we could have several principal main registrations of previous or active treatments. This would complicate the work of the doctor in the clinical tracking of the patient given the difficulty in identifying the main and current register.

Another of the main changes involved the management of the work pending for a specific day and the identification of the related administration programme. In the first version of the PDH, identification of different programmes was managed by events so a patient had a tree of events within their register which represented each of the administrations programmed by the doctor. The new version includes a parameter (closest programme date) calculated automatically on the basis of administration programmes to be carried out by a doctor for a patient.

The final PDH version is described in detail as follows.

### 3.3. Observe & Collect Data

At the stage of implementation, it is important to note recommendations [1] relating to the set- up of the electronic prescription which assists clinical staff in prescribing and, more specifically, in managing the high-risk medication considered in this work.

The PDH attempts to orientate the doctor with prescriptions in that it proposes a dose for each medicine in line with the specific condition of the patient. As such, it minimizes the possibility of errors derived from an incorrect prescription and improves the tracking of the prescribed medication. If the medication selected by the doctor is incorrect, he/she receives an immediate alert to check if indeed that is what is to be prescribed or if it is a human error.

The PDH should function as an information hub which assists in the process of drug administration to patients and, as such, include and automate the process of PVD-PA of medication; it should manage alerts for recommended dosage, elaborate instructions, record the reasons for non-administration of medication, and provide storage and immediate search of lot numbers and expiry dates for both prepared intravenous mixtures and their components for elaboration.

**Figure 1:**
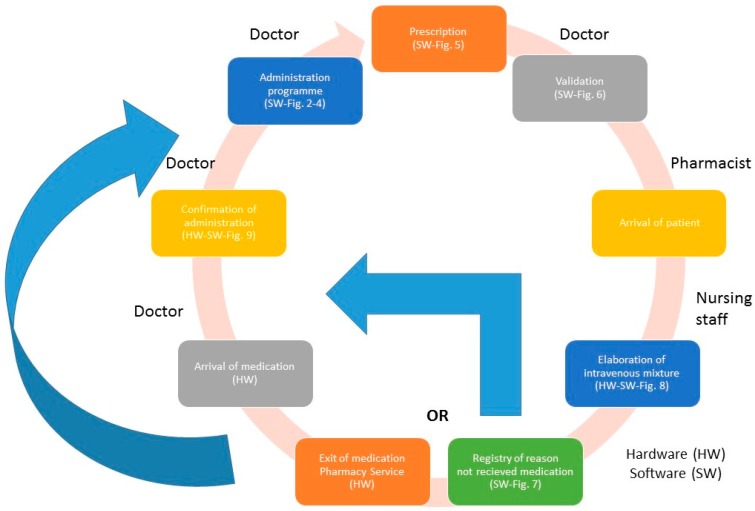
The process of prescription–validation–dosing–preparation–administration (PVD-PA) medication.

The following describes the steps to follow for each phase of the process of medication PVD-PA (Figure 1 represents the process flow).

The PDH should have an intuitive interface with a single view of all the data for each task to complete. The tool should include a module for label printing which can adapt to the information design required by each particular medication to be developed.

A significant number of patients in the PDH suffer from chronic illness and are poly-medicated with the result that they are more vulnerable to potential errors of medication. This protocol allows for, at a single view, the checking of the prescribed medicine, its evolution (in the case where the medicine has already been registered by the doctor), how effective the treatment is, and adherence to it.

**Figure 2:**
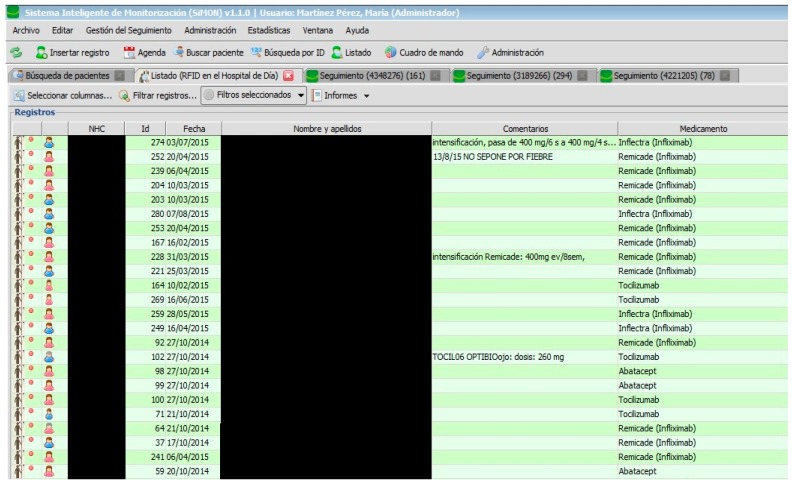
Number of patients.

All users have a start panel where they can consult the list of current patients (see Figure 2).

**Figure 3:**
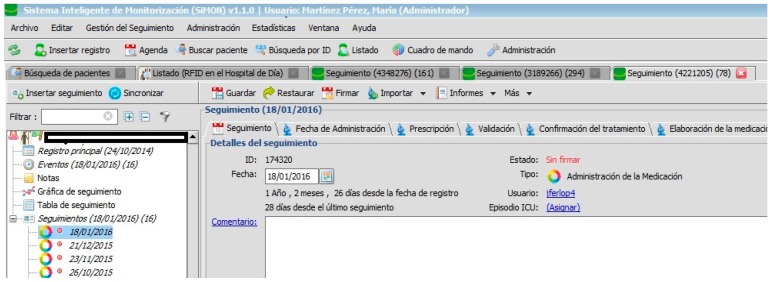
Patient monitoring.

Each patient is associated with a number of monitoring actions and linked to administration programmes devised by the doctor (Figure 3).

**Figure 4:**
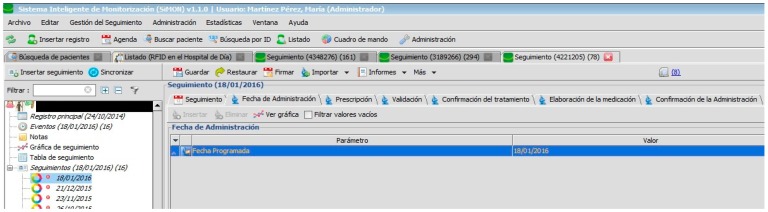
Date of administration programme.

To electronically schedule future administration of treatment, the doctor, firstly, has to create a monitor and introduce a value in the parameter “Programme date” to formalize the new monitoring (see Figure 4).

**Figure 5:**
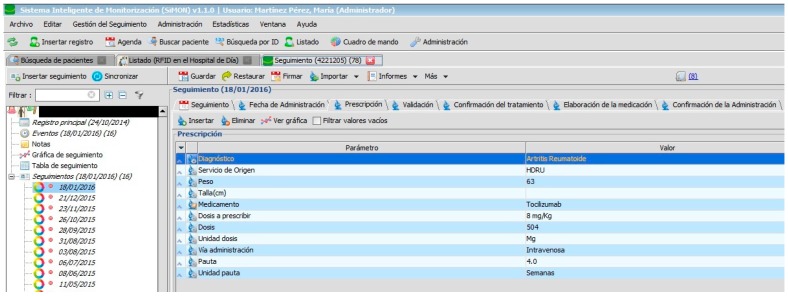
Prescription of medication.

The doctor then has to electronically prescribe the medication as is displayed in Figure 5. The calculation of the recommended dose for the Prescription, Validation, and Elaboration tabs are controlled by rules which establish the correct values for each case with the pharmotherapeutic profile of the patient taken into account.

**Figure 6:**
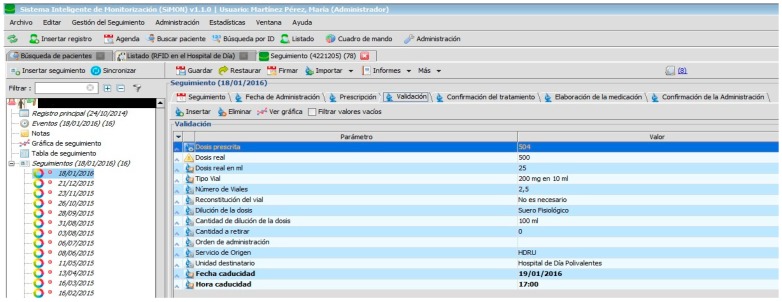
Validation of medication.

The PDH follows the recommendations [1] related to the implementation of systems for electronic prescription which are integrated in the information systems of the hospital and which are used by all those professionals involved in the care process of the patient. In addition, the PDH assists in avoiding the errors most commonly associated with high-risk medication (name of medicine, dosage, schedule, route) as can be seen in Figure 6. It also allows for the use of abbreviations: for example, those used to describe the hospital units involved in the prescription and administration of medication.

Specialist pharmacists in the Hospital Pharmacy can carry out the following tasks as displayed in Figure 6. They can also review the pending tasks, for example, for the present shift, day, week, or any other period of time with the specified treatment or origin of service; if there is a reason for postponement on the part of the administration, they can check the notification that the pharmacist has sent instructing nursing staff to begin preparation of medicine within the protocol. Therefore, a chain of command for work instructions has been established with specific information for each specialist. It is only possible to access and modify clinical information specific to individual health professionals. The PDH also allows for the automation of the dosage, thereby reducing the level of toxicity in medication administered to the patient [1].

**Figure 7:**
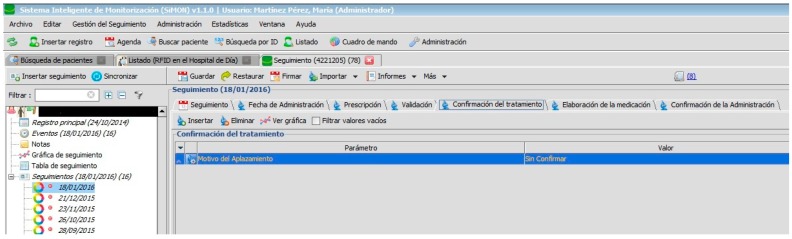
Registry of reasons for patient not receiving medication.

The doctor confirms the health status of the patient and that it is adequate for the administration of medication so that the pharmacist can issue the order for elaboration or, in the opposite case, cancel the programme administration. On the arrival of the patient at the consulting area, the doctor checks that there is no fever and that blood pressure (among other constants) is adequate for the pending treatment (see Figure 7).

This registry assists the doctor in tracking programmes and medication administration and how effective treatment has been in specific pathologies. It is important to systematically revise the medication of chronically ill and poly-medicated patients to detect or prevent adverse incidents, to confirm the adequacy of the medication, and improve adherence to the medication.

**Figure 8:**
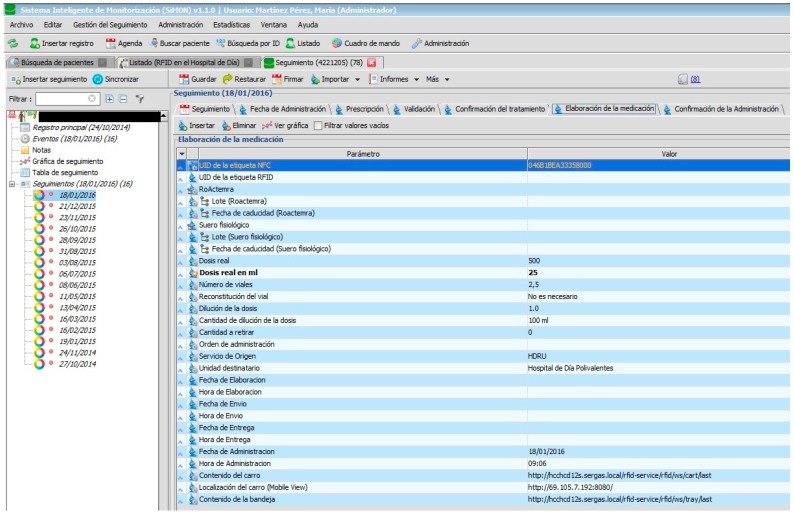
Elaboration of medication.

Nursing personnel, when preparing an intravenous mixture, need to refer to the instructions as shown in the PDH (Figure 8). They can only manage the RFID tag (with the double frequency NFC/UHF) which will identify the elaborated medication and register the lot n° and expiry date of its components. Certain information cannot be changed.

**Figure 9:**
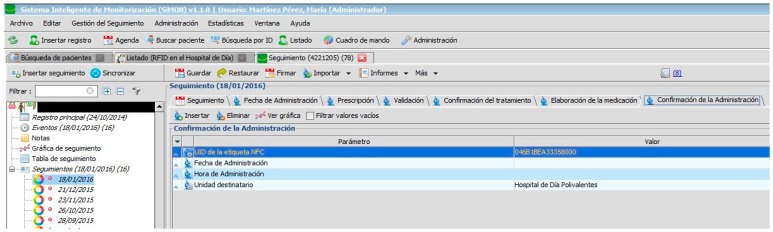
Confirmation of administration.

When the medication is prepared and identified by the RFID tag, it is delivered to the nursing staff of the Day Hospital to be administered to the patient (see Figure 9).

It is important to highlight that approximately 13% of errors recorded in the area of surgery and 67% of those associated with blood-based transfusions are related to the misidentification of patients. The PDH provides measures that guarantee the unambiguous identification of patients, their medication, and clinical information. The PDH follows recommendations for authentic identification in that it uses at least two identifiers (in this case the health service identity card, the RFID tag, and the verbal identity of the patient). This process provides a double automated identity and always verifies the identity of the patient before any procedure.

Nursing staff have to read the identification tag of the patient and medication while the system consults the prescribed and pending medication for that patient. In this way, the possibility for human error is notably reduced.

Training sessions for personnel in the appropriate procedures for identification and verification of a patient were carried out prior, at least before any intervention involving risk. An evaluation and tracking of this identification process has been conducted to detect possible anomalies in the same way as the ownership of the clinical document of the user is during their stay in hospital [1].

### 3.4. Act

The overall result of the system is underpinned by the application previously described with the RFID systems (hardware and software) implemented in the different clinical units of the hospital. Each one of these components is precisely orientated toward fulfilling the care process of the patient on arrival at hospital to receive medication in each of the programmed administrations.

**Figure 10:**
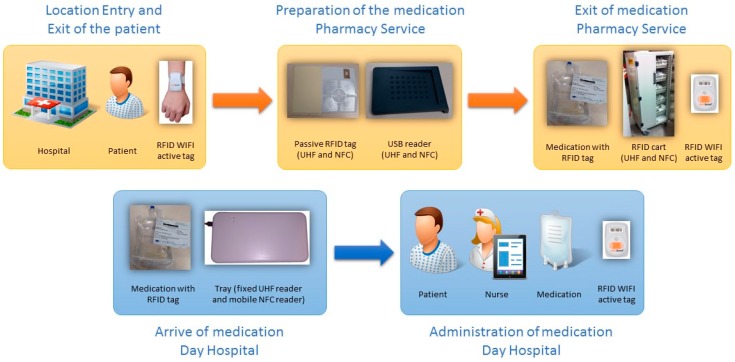
Infrastructure of the radio-frequency identification (RFID) systems.

The RFID systems are the sources of information for the developed application and provide the traceability of patients and medication (location in real time and precise identification) (see Figure 1 and Figure 10):

- Figure 2, Figure 3, Figure 4, Figure 5 and Figure 6 are independent from the hardware infrastructure, showing only the usage of the system software (in terms of time, they are previous to Figure 10).
- Figure 1 and Figure 10 reflect the arrival of the patient at the hospital.
- The preparation of the medication at the Pharmacy Service corresponds to Figure 8 and is represented in Figure 1. It requires both the hardware and software of the system.
- Exit of medication from the Pharmacy Service: this requires hardware and is represented in Figure 1.
- Administration of medication: this requires the system’s hardware and software. Confirmation of administration in Figure 1.
- Arrival of medication: this requires hardware and is represented in Figure 1.

The tracking system of patients is composed of access points and active RFID WIFI components from Aeroscout. The RFID system to trace medication in the Pharmacy service is composed of access points, an RFID cart which reads the medication labelled with double frequency tags (UHF and NFC) and can be located in real time using the RFID WIFI active tag produced by Aeroscout. The RFID system to register the delivery of medication at the Day Hospital (HD) is composed of an RFID tray which registers, in the present application, the time and date of arrival at the HD at the moment the orderly transfers the medication from the cart to the tray. Finally, the RFID system for the administration of medication to the patient and used by the nursing personnel is composed of a mobile telephone with integrated NFC while the medication is tagged using RFID. This RFID system allows the nursing staff to confirm that they are administering the correct medication to the patient and in the proper administration conditions [12].

The main impact of the solution lies with the improved efficiency of the Day Hospital and the Pharmacy Service, improved quality of the tasks, and improved patient security.

The steps to carry out tests have been based on the normal functioning of the Day Hospital service. Training in the correct and safe use of the system has been delivered to health professionals, which is particularly important for treatment involving high-risk medication and poly-medicated patients at all levels of care.

A further objective is to improve the system for notification of errors and incidents involving medication which also includes the evaluation and analysis of the information generated so that measures can be taken for improvement.

It is important to highlight the PDH impact on the increased supervision and vigilance in the safe use of medication in the Pharmacy services and units.

The system improves the identification of patients, standardizes abbreviations, controls high-risk medication, reduces confusion of medicines with similar names, correctly labels all prepared medicines, and foments the active participation of patients in their treatment.

The first tracking to be monitored under this protocol was carried out on the 4th December 2014. A total of 2515 trackings had been conducted by 19th January under the PDH which translates into 2525 administrations to 285 patients.

The importance of the selected readers should be highlighted given that only one approximation is necessary to automatically register the tags. These readers have an emulator keyboard so that no extra action is necessary. A medical computer is integrated into the cabinet with the readers, together with an RFID printer which has an adhesive peel-off label facility which reduces the possibility of contamination. The preparation of these mixtures takes place in sterile conditions so that the computer can verify their sterilization.

## 4. Discussion and Conclusions

The evaluation of this work by the healthcare personnel has been very positive. The system has now been functioning for a number of years and is continually embracing more patients and treatments. Part of this success is due to its design, given that it is very flexible and can meet the new demands that arise in the daily clinical practice of the hospital. The implemented protocol has effectively responded to these needs. The previous PVD-PA process was largely paper- and email-based between health professionals with the essential information often dispersed in different corporate applications.

The most frequent incidents occurred during the first three weeks from the implementation of the protocol. These were detected on reception of the medication in the Day Hospital; approximately 60% of the total was sent without the previous confirmation of administration of treatment by the doctor, as required by the PDH.

In addition, there was the tendency for the Pharmacy Service and the Day Hospital to continue to make telephone calls to ask about the state and progress of the medication (in preparation, transport, administration, the state of health or location of the patient) instead of consulting the relevant information in the established application.

Health personnel have been very collaborative from the beginning and nobody has reacted negatively to the implementation of the project, but one of the greatest concerns related to whether the patient care process would become slower; thanks to the design, this has not been the case. Patients have been very collaborative, although some of them had doubts as to how the process worked. During the first eight months, the contracted computer engineer attended the Day Hospital three times a week to resolve patients’ doubts and distribute information brochures. In general, patients were content with the reduced waiting times.

The implemented protocol, in conclusion, has effectively responded to the needs of health professionals with respect to the tracking of patients and medication in daily clinical practice during the process of prescription–validation–elaboration–dispensation–administration of intravenous mixtures to patients in the Day Hospital. In addition, the system facilitates the emission of routine reports and the use of data from the pharmatechnical section of the Pharmacy Service (for example, the number of medication units consumed on a monthly basis by a particular service). It is important to note that 2525 administrations prescribed to 285 patients have been tracked. This medication includes Abatacept, Tocilizumab, Remicade (Infliximab), and biosimilars such as Inflectra (Infliximab). These treatments have been prescribed by doctors for pathologies belonging to the CHUAC Digestive Health, Dermatology, and Rheumatology services.

This project is considered highly innovative in its field and applications now exist in the market which integrate the functionalities that have been described, including RFID technology, allowing for reduced times in learning and development for health service personnel. It is interesting to note the capacity for adaptability, modification, and scaleability of all parameters in its elements, whether they are being used by doctors, patients, pharmacists, chemists, or nursing staff in the Pharmacy or Day hospital. In addition, the method functions almost in real time to include new medication, patients, modifications of schedules, prescriptions, and updates in administration programmes.

An imminent consideration, with the view to incorporating the remaining biological therapies, is the development of a BI (Business Intelligence) [25] which would allow for a greater use of data responding to most of the information needs of the management of the hospital.

Future work will include integrating into the service, using other existing protocols, other medications called biological therapies that are considered high-risk and expensive, so that their tracking, from the time of elaboration, prescription, conservation, transport conditions, stability, etc., to the registering of the prescription parameters (medication, dosage, regimen) can be assured.

## References

[1] Estrategia de Seguridad del Paciente del Sistema Nacional de Salud Período 2015–2020. https://www.seguridaddelpaciente.es/resources/documentos/2015/Estrategia%20Seguridad%20del%20Paciente%202015-2020.pdf.

[2] Documento de ayuda a la implantación de un sistema de Trazabilidad en la distribución de medicamentos. www.aecoc.es.

[3] Davis S. (2004). Tagging along. RFID helps hospitals track assets and people. Health Facil. Manag..

[4] Christe B., Cooney E., Maggioli G., Doty D., Frye R., Short J. (2008). Testing potential interference with RFID usage in the patient care environment. Biomed. Instrum. Technol..

[5] Iadanza E., Dori F., Miniati R., Bonaiuti R. Patients tracking and identifying inside hospital: A multilayer method to plan an RFID solution. Proceedings of the 30th Annual International Conference of the IEEE Engineering in Medicine and Biology Society.

[6] Kim D., Kim J., Kim S., Yoo S. Design of RFID based the Patient Management and Tracking System in hospital. Proceedings of the 30th Annual International Conference of the IEEE Engineering in Medicine and Biology Society.

[7] Martínez-Pérez M., Vizoso J., Bello E., Broullón J., Penas A. Modelado del proceso de prescripción-dispensación-administración de medicamentos en el Complejo Hospitalario Universitario Juan Canalejo. Proceedings of the Sociedad Española de Informática de la Salud, XI Congreso Nacional de Informática de la Salud Inforsalud 2008.

[8] Martínez-Pérez M., Vizoso J., Carrajo L., Rimada A., Lamelo A., Llamas D., Vázquez-González G. Cómo utilizar RFID en el ámbito sanitario. Proceedings of the Aplicabilidad en la obtención de la trazabilidad de pacientes y la prevención de eventos adversos. En The Spanish Association for Tracking and Tracing (Eds.), Terceras jornadas sobre RFID.

[9] Martínez Pérez M., Cabrero-Canosa M., Vizoso Hermida J., Carrajo García L., Llamas Gómez D., Vázquez González G., Martín Herranz I. (2012). Application of RFID Technology in Patient Tracking and Medication Traceability in Emergency Care. J. Med. Syst..

[10] Dafonte C., Castro A., Gómez A., Arcay B. (2007). Intelligent Agents Technology Applied to Task Scheduling and Communications Management in a Critical Care Telemonitoring System. Comput. Biol. Med..

[11] Xerencia de Xestión Integrada A Coruña. http://hospitalcoruna.sergas.es/conocenos/Informaciondocomplexo/localizacion/Paginas/HospitalACoruna.aspx.

[12] Martínez Pérez M., Vázquez González G., Dafonte C. (2016). Safety and Traceability in Patient Healthcare through the Integration of RFID Technology for Intravenous Mixtures in the Prescription-Validation-Elaboration-Dispensation-Administration Circuit to Day Hospital Patients. Sensors.

[13] Martínez Pérez M., Vázquez González G., Dafonte C. (2016). Evaluation of a Tracking System for Patients and Mixed Intravenous Medication Based on RFID Technology. Sensors.

[14] Huang Y., Chu C., Lin Y., Kuo C. RFID Applications in Hospitals—A Case Study for Emergency Departments. Proceedings of the 16th International Conference on Distributed Multimedia Systems, DMS 2010.

[15] Wessel R. (2007). Czech Hospital Using HF RFID to Track Chemotherapy Drugs. http://www.rfidjournal.com/article/view/3394.

[16] Wu F., Kuo F., Liu L.-W. The application of RFID on drug safety of inpatient nursing healthcare. Proceedings of the 7th International Conference on Electronic Commerce (ICEC’05).

[17] RFID Journal. http://www.rfidjournal.com/healthcare.

[18] García Suárez B. El control de ‘Stock’ por RFID llega al Hospital con Promesa de Fiabilidad, 2010. http://www.correofarmaceutico.com/2010/10/11/gestion/control-de-stock-por-rfid-llega-hospital-con-promesa-de-fiabilidad.

[19] Amini M., Otondo R., Janz B., Pitts M. (2007). Simulation modeling and analysis: A collateral application and exposition of RFID technology. Prod. Oper. Manag..

[20] Janz B.D., Pitts M.G., Otondo R.F. ‘Back to future with RFID, lessons learned-some old, some new’. Proceedings of the Workshop, the 35th Annual Meeting of the Decision Sciences Institute.

[21] Darianian M., Michael M.P. A low power pervasive RFID identification system for medication safety in hospital or home Telecare. Proceedings of the 3rd International Symposium on Wireless Pervasive Computing (ISWPC 2008).

[22] Lai C.L., Chien S.W., Chang L.H., Chen S.C., Fang K. Enhancing medication safety and healthcare for inpatients using RFID. Proceedings of the 7th Portland International Center for Management of Engineering and Technology Conference (PICMET’07).

[23] Sun P.R., Wang B.H., Wu F. (2008). A new method to guard inpatient medication safety by the implementation of RFID. J. Med. Syst..

[24] Corbal-Ramón G.I., Cabrero-Canosa M., Carrajo L., Vázquez O., Rimada L., Vázquez G. (2010). Co-morbidity Analysis And Decision Support on Transplanted Patients using Machine Learning Techniques. Medical and Care Compunetics.

[25] Vercellis C. (2011). Business Intelligence: Data Mining and Optimization for Decision Making.

